# mTOR pathway in familial focal epilepsies

**DOI:** 10.18632/oncotarget.14234

**Published:** 2016-12-26

**Authors:** Théo Ribierre, Stéphanie Baulac

**Affiliations:** ^1^ Sorbonne Universités, UPMC Univ Paris 06, Inserm, CNRS, APHP, Institut du Cerveau et la Moelle (ICM), Hôpital Pitié-Salpêtrière, Paris, France

**Keywords:** mTORC1, DEPDC5, focal epilepsy, focal cortical dysplasia, rapamycin, Neuroscience

An increasingly recognized role of genes encoding components of the mammalian Target Of Rapamycin (mTOR) signal transduction pathway has recently emerged among familial focal epilepsies. Loss-of-function mutations in the Dishevelled, Egl-10 and Pleckstrin Domain Containing protein 5 (*DEPDC5*) gene, and lately in the genes encoding Nitrogen Permease Regulator Like 2 and 3 proteins (NPRL2 and NPRL3), have been revealed in families with inherited focal epilepsies [1, 2]. DEPDC5 protein, together with NPRL2 and NPRL3, form the GAP Activity TOwards Rag complex 1 (GATOR1), which acts as a negative regulator of the mTOR Complex 1 (mTORC1) [3]. This complex regulates essential functions in the cell, including proliferation and growth, through the modulation of transcriptional and translational activity. Mutations in GATOR1-encoding genes lead to hyperactivity of the mTORC1 pathway, highlighting mTORopathies as a novel pathomechanism to a field so far dominated by ion channelopathies.

*DEPDC5* mutations are to date the most common cause of familial focal epilepsies. Mutations are associated with diverse focal epileptic phenotypes ranging from apparently nonlesional focal epilepsies to malformation-associated focal epileptic syndromes, which include Focal Cortical Dysplasia (FCD) and hemimegalencephaly (review [4]). Distinct phenotypes may be observed among patients from the same family. While there is still no evidence to explain what underlies this phenotypic variability within related patients, one possible scenario is the occurrence of brain somatic mutations [5]. Recently, this hypothesis was confirmed with the identification of a second-hit somatic mutation over a germline mutation of *DEPDC5* in the resected brain tissue of an FCD patient presenting with familial inherited epilepsy [6]. The discovery of mutations in genes encoding proteins of the mTORC1 pathway in FCD represents a major breakthrough clarifying the long-standing observations of mTORC1 constitutive activation in dysmorphic neurons (FCD type IIa) and balloon cells (FCD type IIb) present in patient tissues.

Lately, Marsan *et al*. reported the first animal model of the pathology: a *Depdc5* knockout rat [7]. Noteworthy, homozygous knockout embryos died as early as E14.5 from global growth delay. Heterozygous knockout animals displayed constitutive mTORC1 activation, abnormal cortical neuron firing patterns, as well as cortical dysmorphic neurons and balloon-like cells, evocative of FCD type IIb. However, these changes were not associated with spontaneous electroclinical seizures. Prenatal treatment with an mTORC1 inhibitor, rapamycin, rescued the embryonic lethal phenotype of homozygous knockout animals and prevented the development of the brain pathology in heterozygous animals. In summary, *Depdc5*-knockout rats exhibited several features of rodent models of mTORopathy, demonstrating that the pathogenic mechanism of *DEPDC5*-related epilepsies is clearly associated to an upregulation of the mTORC1 pathway.

One of the upcoming challenges will be to elucidate why mutations in a gene encoding a ubiquitously expressed protein lead to purely neurological symptoms with focal seizures and/or lesions. In sharp contrast, tuberous sclerosis, a well-characterized multisystem disease with abnormal brain development and intractable seizures, is due to mutations in either of the two genes (*TSC1* and *TSC2*) encoding proteins of the Tuberous Sclerosis Complex (TSC), another negative regulator of mTORC1 [8]. Future studies should therefore be directed towards the investigation of the pathophysiological mechanisms specifically at stake in GATOR1-related focal epilepsies. First, interrogating the brain function of DEPDC5 is a compelling start point to understand the broad - although neurological - phenotypic spectrum associated with *DEPDC5* mutations. Second, since mTORC1 is known to regulate gene transcription and protein translation impaired mTORC1 activity could consequently lead to abnormal gene expression profiles and aberrant protein synthesis. Thus, an interesting line from this point of view would be to explore divergent transcriptional profiles in patient resected brain tissue, with an emphasis on ion channels and neurotransmitters levels of expressions, in order to link mTORopathies to disturbed neuronal activity.

Overall, *DEPDC5* mutations are the most frequent cause in familial focal epilepsies, and differ from any other mutations in ion channel encoding genes by the presence of cortical malformations. The generation of relevant *in vivo* and *in vitro* models is thus critical to better characterize the molecular mechanisms involved. At last, efforts will be directed towards the identification of new therapeutic approaches and drug selection to improve drug-resistant patient prognosis.
